# Sideburns Obesity Syndrome: Is Sideburn Fat the Window to the Heart?

**DOI:** 10.7759/cureus.12706

**Published:** 2021-01-14

**Authors:** Mohammed Abrahim

**Affiliations:** 1 Family Medicine, McMaster University, Hamilton, CAN; 2 Emergency Department, Halton Healthcare, Milton, CAN

**Keywords:** sideburns, obesity, cardiovascular, metabolic syndrome, facial fat, frank’s sign, bichat fat pad, buccal fat pad, visceral fat percentage, physical exam

## Abstract

Despite significant advancements in cardiovascular diagnostic technology, physical examination signs remain credible diagnostic indicators of coronary artery disease. Here, we report a case of a 50-year-old male patient with premature coronary artery disease associated with a novel physical sign of bilateral symmetrical bulging of the sideburn areas of the face. The sideburns correspond anatomically to the buccal fat pad which is composed of visceral adipose tissue. Visceral obesity is an established risk factor for cardiovascular disease, independent of total body weight, with some suggestion of a causal association. Therefore, isolated buccal visceral obesity (sideburn obesity syndrome) could be a marker for premature coronary artery disease.

## Introduction

Visceral obesity is strongly associated with cardiovascular disease risk, with a growing body of research demonstrating a potential causal association [1]. Visceral fat depots are not limited to the abdomen, they also extend into the chest (epicardial), blood vessels (perivascular), and the face (buccal) [2]. Despite being anatomically separate, the buccal fat pad (BFP) and abdominal visceral adipose tissue appear to be histologically and metabolically identical and, because of this, the BFP is sometimes referred to as the visceral fat of the face [2]. The BFP was first described in 1801 by the French anatomist Xavier Bichat as an anatomically distinct, deep biconvex encapsulated mass of adipose tissue that is located between the masticatory muscles [3]. BFP has a triangular shape with a caudal base and can be up to 4 cm in size [4]. The surface anatomy of the BFP corresponds to the lateral aspect of the face at precisely the sideburn areas and bounded superiorly by the zygomatic bone, the mandibular margin inferiorly, the zygomaticus major muscle anteriorly, and the anterior tragal line posteriorly. In overweight adults, the size of the main part of the BFP, obtained from ultrasound measurements, was significantly correlated with all anthropometric parameters such as total body weight and body mass index (BMI) [5]. Levine et al. used randomized and blinded CT scans of the head and abdomen to measure the association between the size of the buccal and abdominal visceral fat. The results indicated that the size of abdominal visceral adipose tissue was strongly associated with buccal adipose tissue. This relation appears to be specific to visceral fat rather than general adiposity [6]. One large Korean study measured multiple facial anthropometric dimensions and compared them with visceral obesity and concluded that the distance between the lateral sides of the face (at the sideburns areas) as measured between the inferior earlobes was the strongest predictor of visceral obesity [7].

## Case presentation

A 50-year-old Canadian male of south-Asian descent with a history of premature coronary artery disease presented to our Emergency Department with an episode of resolved anginal chest pain. Five years prior to his visit, he sustained an acute anterior ST-elevation myocardial infarction (STEMI) at 45 years of age, for which he underwent a successful primary angioplasty of the proximal left anterior descending (LAD) artery with a drug-eluting stent. He was also placed on dual antiplatelet therapy. At the time of his STEMI, he had mild non-familial dyslipidemia without type 2 diabetes, hypertension, or any other medical conditions. In terms of lifestyle risk factors, he reported being an ex-smoker, having quit 15 years earlier at the age of 35. He undertook average levels of physical activity while reporting a diet that consisted of a traditional high-fat south-Asian diet. He also had a strong cardiac family history, his father and brother both suffered premature coronary artery disease.

**Figure 1:**
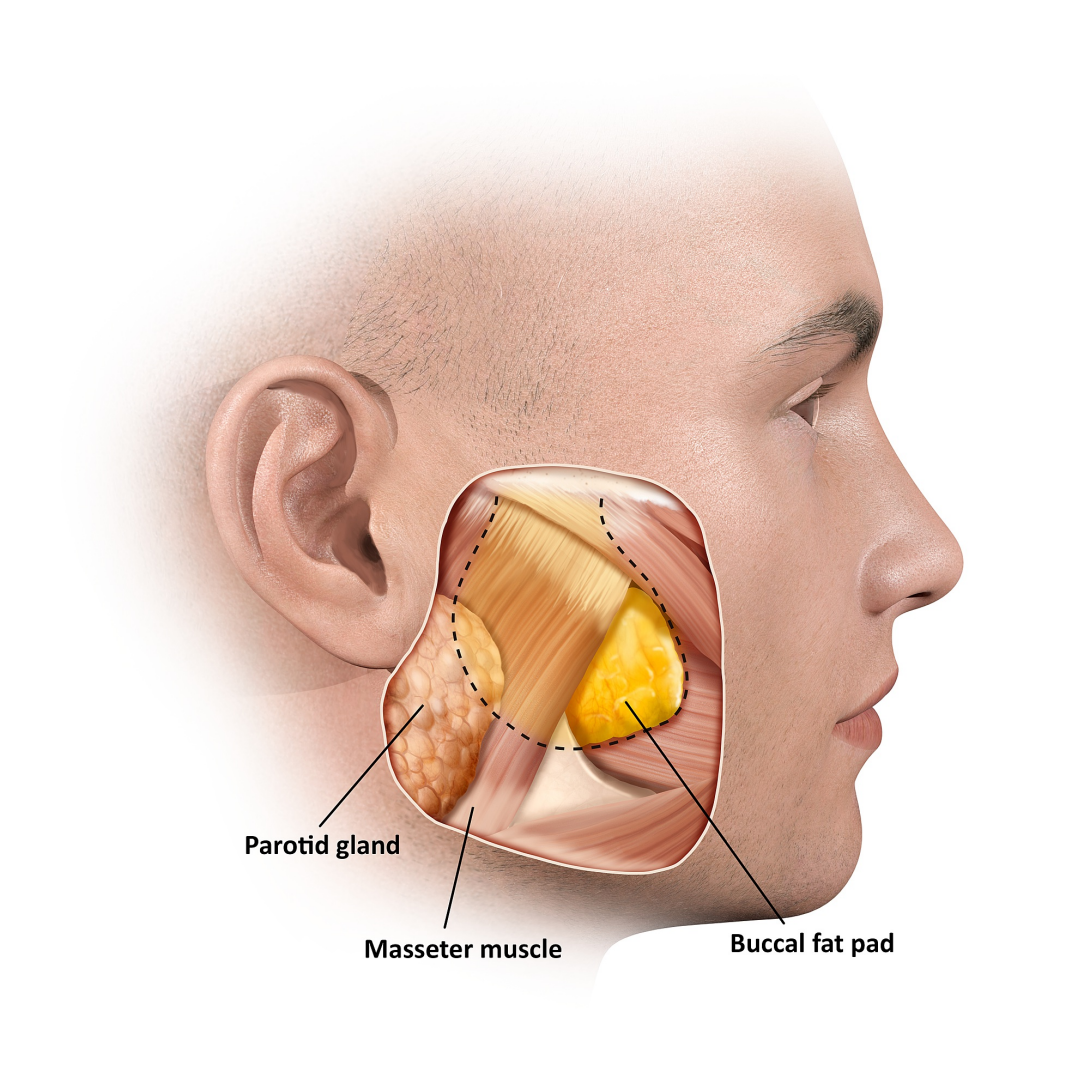
Buccal (Bichat) fat pad corresponding to the sideburn obesity sign Anatomical illustration of the underlying structures of the sideburn area of the face

**Figure 2:**
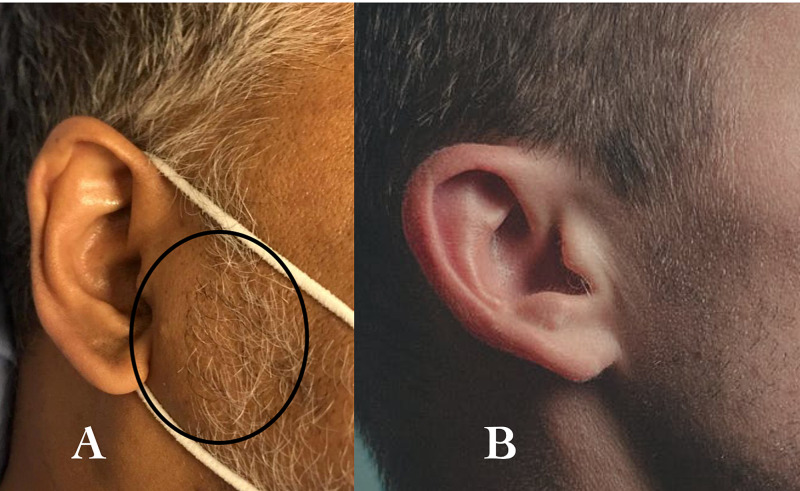
(A) The reported case with circled sideburn obesity (B) Normal sideburn area

The physical examination revealed normal vital signs, no overt signs of obesity with normal body weight (BMI of 24.7 kg/m2) and normal waist circumference. The cardiovascular physical exam was also unremarkable. We noted that the patient appeared to have bilateral symmetrical localized areas of facial bulge corresponding to the buccal fat pad (Figure 1) of the sideburn area of the face with normal cheeks (Figure 2). When asked about the area of interest, the patient mentioned two interesting aspects: 1) that he had always had it, and 2) that his father and brother also had noticeable sideburn fat. His serial electrocardiograms (ECGs) demonstrated normal sinus rhythm and old q waves in leads v1 and v2 with no dynamic changes. His laboratory investigations were within normal limits. 

## Discussion

The case reported above exhibited bilateral obesity within the sideburn region of the face and premature coronary artery disease. Although a single case report cannot provide a clear demonstration of an association and further research is needed to confirm such an association and offer the etiopathogenesis of the preferential fat deposition in the BFP. Three possible explanations offer themselves: (i) Ethnicity: south-Asian men may have a relatively greater tendency for buccal visceral fat obesity which might be a marker of their relatively high cardiovascular risk [8]; (ii) Dietary: high-fat diet such as the south-Asian diet reported by the patient could lead to preferential deposition in the visceral compartment; and (iii) Hormonal: the androgen/estrogen (A/E) ratio plays a significant role in adipose tissue distribution. A high E/A ratio favors visceral fat deposits and gives the body the android shape found among adult men and postmenopausal women [9]. The three proposed explanations could occur independently of each other or in a complex interplay. Because assessing visceral obesity in clinical practice is usually performed via measuring waist circumference (WC), this could be challenging due to the inclusion of the subcutaneous adipose tissue and intra-intestinal contents. The accuracy of WC measurement is dependent on the practitioner's experience and patients' cooperation. However, visual inspection of the sideburns areas of the face could give a subtle impression of the size of visceral adipose tissue. Visceral obesity of the BFP was also suggested as a cause of other facial physical signs that are linked to cardiovascular risk - including preauricular creases and the diagonal earlobe crease (Frank’s sign) [10]. 

## Conclusions

Increased visceral fat is a strong predictor of premature cardiovascular disease. The buccal fat pad is compositionally identical to visceral adipose tissue. Here we present a case of isolated bilateral obesity of the sideburns area of the face associated with premature coronary artery disease. The authors suggest that the sideburns obesity sign corresponds to, and may be associated with, premature coronary artery disease risk. Further research is needed to confirm the association suggested herein and identify rates of the sideburn obesity sign within the general population. Additionally, the mechanism of the preferential fat deposition in the sideburn areas of the face is yet to be explored.
